# Health-Related Quality of Life after Pediatric Traumatic Brain Injury: A Quantitative Comparison between Children’s and Parents’ Perspectives of the QOLIBRI-KID/ADO Questionnaire

**DOI:** 10.3390/jcm12237439

**Published:** 2023-11-30

**Authors:** Katrin Cunitz, Ivana Holloway, Anne Harzendorf, Sven Greving, Marina Zeldovich, Ugne Krenz, Dagmar Timmermann, Inga K. Koerte, Michaela Veronika Bonfert, Steffen Berweck, Matthias Kieslich, Knut Brockmann, Maike Roediger, Anna Buchheim, Nada Andelic, Michael Lendt, Michael Staebler, Holger Muehlan, Nicole von Steinbuechel

**Affiliations:** 1 Institute of Psychology, University of Innsbruck, Universitaetsstr. 5-7, 6020 Innsbruck, Austria; marina.zeldovich@uibk.ac.at (M.Z.); anna.buchheim@uibk.ac.at (A.B.); nicole.von-steinbuechel@uibk.ac.at (N.v.S.); 2 Department of Psychiatry and Psychotherapy, University Medical Center Goettingen, Von-Siebold-Str. 5, 37075 Goettingen, Germany; 3 Institute of Medical Psychology and Medical Sociology, University Medical Center Goettingen, Waldweg 37A, 37073 Goettingen, Germany; ivana.holloway@med.uni-goettingen.de (I.H.); anne.harzendorf@med.uni-goettingen.de (A.H.); sven.greving@med.uni-goettingen.de (S.G.); ugne.krenz@med.uni-goettingen.de (U.K.); dagmar.timmermann@med.uni-goettingen.de (D.T.); 4 cBRAIN, Department of Child and Adolescent Psychiatry, Psychosomatics, and Psychotherapy, LMU University Hospital, Ludwig-Maximilian University, Nussbaumstrasse 5, 80336 Munich, Germany; inga.koerte@med.uni-muenchen.de; 5 Department of Pediatric Neurology and Developmental Medicine, LMU Center for Development and Children with Medical Complexity, Dr. Von Hauner Children’s Hospital, LMU University Hospital, Haydnstr. 5, 80336 Munich, Germany; michaela.bonfert@med.uni-muenchen.de; 6 Specialist Center for Paediatric Neurology, Neurorehabilitation and Epileptology, Schoen Klinik, Krankenhausstraße 20, 83569 Vogtareuth, Germany; sberweck@schoen-klinik.de; 7 Department of Paediatric Neurology, Hospital of Goethe University, Theodor-Stern-Kai 7, 60590 Frankfurt am Main, Germany; matthias.kieslich@kgu.de; 8 Interdisciplinary Pediatric Center for Children with Developmental Disabilities and Severe Chronic Disorders, Department of Pediatrics and Adolescent Medicine, University Medical Center, Robert-Koch-Str. 40, 37075 Goettingen, Germany; knut.brockmann@med.uni-goettingen.de; 9 Department of Pediatrics and Adolescent Medicine, General Pediatrics, Intensive Care Medicine and Neonatology, University Hospital Muenster, Albert-Schweitzer-Campus 1, 48149 Muenster, Germany; maike.roediger@ukmuenster.de; 10 Research Centre for Habilitation and Rehabilitation Models and Services (CHARM), Department of Health and Society, University of Oslo, 0313 Oslo, Norway; nadaan@uio.no; 11 Department of Physical Medicine and Rehabilitation, Oslo University Hospital, 0424 Oslo, Norway; 12 Neuropediatrics, St. Mauritius Therapeutic Clinic, Strümper Straße 111, 40670 Meerbusch, Germany; lendt@stmtk.de; 13 Hegau-Jugendwerk GmbH/Neurological Rehabilitation Center for Children, Adolescents and Young Adults, Kapellenstr. 31, 78262 Gailingen am Hochrhein, Germany; michael.staebler@hegau-jugendwerk.de; 14 Department of Health and Prevention, University of Greifswald, Robert-Blum-Str. 13, 17487 Greifswald, Germany; holger.muehlan@uni-greifswald.de

**Keywords:** disease-specific health-related quality of life, QOLIBRI-KID/ADO, traumatic brain injury, children and adolescents, self- and proxy reports

## Abstract

Pediatric health-related quality of life (HRQoL) as a measure of subjective wellbeing and functioning has received increasing attention over the past decade. HRQoL in children and adolescents following pediatric traumatic brain injury (pTBI) has been poorly studied, and performing adequate measurements in this population is challenging. This study compares child/adolescent and parent reports of HRQoL following pTBI using the newly developed Quality of Life after Brain Injury in Children and Adolescents (QOLIBRI-KID/ADO) questionnaire. Three hundred dyads of 8–17-year-old children/adolescents and their parents were included in the study. The parent–child agreement, estimated using intraclass correlation coefficients and Cohen’s κ, displayed poor to moderate concordance. Approximately two-fifths of parents (39.3%) tended to report lower HRQoL for their children/adolescents on the total QOLIBRI-KID/ADO score. At the same time, about one-fifth (21.3%) reported higher HRQoL Total scores for their children/adolescents. The best agreement for parents rating adolescents (aged 13–17 years) was found in terms of the Total score and the Cognition and Self scale scores. To date, parent-reported HRQoL has been the preferred choice in pediatric research after TBI. However, with a parent–child disagreement of approximately 60%, our results highlight the importance of considering self-reports for children/adolescents capable of answering or completing the HRQoL measures.

## 1. Introduction

The consequences of pediatric traumatic brain injury (pTBI) present a challenge in nearly every population and demographic group [1], as TBI is a leading cause of hospitalization in children and adolescents in Germany [2]. In the past, research has largely focused on measuring the impact of TBI on the lives of children and adolescents using classical outcome measures, such as physical functioning, mortality rates, overall disability scores, or neuropsychological functioning [3]. The subjectively perceived well-being of the child itself is often overlooked [4]. However, many children and adolescents after TBI still suffer from short- and long-term functional [5], cognitive [6,7], emotional [8], and social impairments [7,9]. Recently, the development and application of patient-reported outcome measures (PROMs) in different health domains has received considerable attention, especially concerning health-related quality of life (HRQoL) [10]. HRQoL comprises an individual’s satisfaction with different life domains, including physical, emotional, mental, social, daily life, and behavioral components [11]. The HRQoL construct is used as an outcome indicator of subjective well-being after illness, injury, and treatment [12,13]. Assessing HRQoL is important for patients, as the experience of illness and treatment goes far beyond physiological consequences and objective outcomes [4]. 

Several generic and disease-specific instruments have been developed for assessing HRQoL. Generic instruments collect information in both healthy and affected individuals and allow comparisons to be drawn between populations, irrespective of health conditions [14]. Specific HRQoL measures are designed to capture specific diseases or interventions and therefore tend to be more sensitive, e.g., to detect changes in treatment [14]. In the field of pTBI, no disease-specific HRQoL instrument has been developed to date, e.g., [15]. Recently, however, the psychometric characteristics of a new pTBI-specific instrument, the Quality of Life after Brain Injury in Children and Adolescents (QOLIBRI-KID/ADO), have been published [16].

Ideally, HRQoL after pTBI should be self-reported by the children and adolescents concerned [17] to determine their subjective well-being and functioning [18], as they are the best experts for their subjective health [12]. However, in most cases parental reports are used in pTBI research [17,19]. As there are often large differences between self-reports and parental reports, e.g., [20], convergences or systematic differences in the information provided by children/adolescents and their parents regarding HRQoL need to be thoroughly investigated. 

Methodologically, one way of obtaining data from the proxy perspective is the so-called proxy–patient perspective, in which proxies are asked how they think the patient would respond [21]. In most cases, this perspective is applied as a surrogate for self-assessment, but it may entail various biases; proxies may underestimate certain domains of health and unobservable domains, such as emotions [21]. Parental reports should therefore be interpreted with caution and with the understanding that parents may assess the HRQoL of their children differently [22] from the self-reports of the children concerned. Research on children aged five years and older indicates their general ability to reliably answer questions about their HRQoL when age-appropriate measures are administered, e.g., [23]. 

In pediatric populations, regardless of health status, there is no clear evidence with regard to the level of concordance between parents’ and their children’s/adolescents’ reports on HRQoL. Agreement ranges from poor, e.g., [20,24], to moderate, e.g., [25,26], good, e.g., [27,28], or even excellent [29], depending on the specific populations, measures, or subscale dimensions, e.g., [15,30]. After pTBI, children/adolescents and their parents do in fact prioritize different aspects of HRQoL. Parents appear to focus more on behavioral problems (e.g., hyperactivity, conduct behavior, and peer problems [31,32]), social impairments [33], complaints about existing cognitive dimensions [33], and paying more attention to limitations in daily functioning [31]. Children and adolescents are more likely to emphasize psychosocial issues related to their HRQoL, e.g., [34], such as the importance of social activities [31], school functioning [35], or post-injury changes in skills, personality, and behavior [34].

Despite the increasing number of studies on HRQoL after TBI, factors that may influence parent–child agreement are less commonly investigated. The age of children and adolescents at the time of the TBI may be an important factor, since different developmental milestones are reached at different ages [36]. The studies on the age-related influence on parent–child agreement after pTBI are sparse. Furthermore, the age-related interpretation of dis-/agreement is ambiguous, and several explanations are possible: compared to older children, the agreement between younger children and their parents has been reported to be higher, possibly because parents spend more time with them [37]. Conversely, agreement has also been reported to be lower, perhaps due to difficulties experienced by younger children in expressing their needs [37,38,39]. Studies reported moderate to strong agreement between parents and healthy adolescents, although it decreased with increasing age [25,40]. In addition, the assessment of the HRQoL dimension should be considered; the older the adolescents are, the greater the disagreement seems to be, especially in the physical and psychosocial domains [25,29], whereas the social relation domain showed only fair agreement during adolescence [25]. A recent review reported that age-related results for different health conditions are also ambiguous [41]. The authors of the review summarized that no age effect or discrepancies were explained in terms of the different perspectives of parents and children on health issues [41]. Some studies reported an increasing parent–child agreement in children with a chronic illness [23], epilepsy [42], and asthma [43] due to improved communication skills with age or by overlooking the perspective of very young children,. However, other studies reported lower agreement with increasing age in children with cystic fibrosis [44] and cancer [45], especially with greater differences in physical, psychological, social, and cognitive subscales [45]. This has been explained by the fact that parents can only provide limited information for children aged eleven years and older because the children are becoming more independent from their parents [45]. The few studies of C&A after TBI have shown the same ambiguous patterns, either reporting a decrease in agreement with age, explained by the fact that adolescents seeking more independence may be less willing to disclose symptom status to their parents [40,46], either non-influence of severity or ren after or authors found no age effects [20,26]. 

There is an ongoing discussion as to whether the level of severity of health issues might be a moderator of the agreement [47]. Studies looking at the agreement between reports have described either no impact, e.g., [26], or an impact, e.g., [46], of different levels of pTBI severity. Reports by adolescents after severe TBI were in greater agreement with those of their parents than those by adolescents after mild and moderate TBI, especially on physical, social, and psychosocial subscales [28]. This has been explained by a greater dependency on the caregiver after severe TBI, e.g., [37]. Contrarily, a study assessing the awareness of problems in the daily life, interpersonal, cognitive, and emotional domains found that C&A after severe TBI underestimated their competence, whereas responses of C&A after mild and moderate TBI were more consistent with the proxy informants [48]. A recent review notes that adolescents after moderate and severe TBI are more likely to have impaired awareness of their deficits in, for example, memory or communication skills [49], which is associated with lower self-esteem [50] and with poorer parent–child agreement on aspects such as child adaptive functioning and emotional and behavioral problems [51].

In addition, factors such as sex, recovery, the presence or absence of chronic health conditions, and pre-TBI mental health problems of the child/adolescent may also influence self- and parent reports and their agreement. Research into adult TBI has shown that females tend to rate their subjective well-being significantly lower than males [52]. This may also be true in children, meaning that HRQoL is poorer in girls than in boys [26,46]. To the best of our knowledge, however, there is a paucity of literature on the impact of sex, post-concussion symptom burden, comorbidities, and mental health issues on parent–child agreement. 

The aim of the current study is, therefore, to assess the degree of agreement between self-reported disease-specific HRQoL of children and adolescents after TBI and the parental proxy–patient perspective, using the newly developed QOLIBRI-KID/ADO questionnaire [16]. The impact of age, TBI severity, sex, recovery, and the presence of chronic or mental health diseases on the agreement between children, adolescents, and their parents was also investigated. Based on the literature, we expect parents to underestimate their children’s and adolescents’ HRQoL, e.g., [3,35]. We also expect a decrease in agreement with increasing age, e.g., [46], and with the severity of TBI, e.g., [49]. 

## 2. Materials and Methods

### 2.1. Study Sample

For this retrospective, cross-sectional, multicenter study, a convenience sample consisting of 300 participants and their parents was recruited from 15 hospital registries in Germany from January 2019 to January 2022.

Participants were included if they (a) were eight to 17 years of age, (b) had a diagnosis of TBI at least three months to ten years prior to study enrollment, (c) had a formal Glasgow Coma Scale (GCS [53]) score or recorded TBI severity, (d) were outpatients (or about to resume inpatient treatment), and (e) were able to understand and answer the questions. 

Participants were excluded if they were currently in a vegetative state, or had spinal cord damage, a severe mental illness before TBI (e.g., psychosis, autism, etc.), epilepsy before TBI, a terminal disease (e.g., advanced cancer or advanced heart disease), or very severe poly-trauma (characterized by multiple, simultaneous injuries to different parts of the body, one of which or a combination of which was life threatening) impacting the HRQoL more than the TBI.

The diagnoses and/or clinical description in the medical records were extracted and children were included or excluded based on the inclusion/exclusion criteria before the recruiting clinic staff mailed the invitations to the potential participants. The children and their families were informed about the research aims and procedure before giving their written consent. All participants and their parents/legal guardians signed the informed consent.

### 2.2. Measures and Data Collection

The QOLIBRI-KID/ADO [16] is a novel TBI-specific, age-adapted HRQoL questionnaire for children (KID: aged 8–12 years) and adolescents (ADO: 13–17 years) after TBI. The questionnaire is developed as self- and proxy versions. The items refer to the last week or the present and are self- or proxy-rated on a five-point scale (“not at all”, “slightly”, “moderately”, “quite”, “very”) with higher scores indicating higher HRQoL. The questionnaire consists of 35 items assigned to six domains, with four domains measuring satisfaction with Cognition (seven items), Self (five items), Daily Life and Autonomy (seven items), and Social Relationships (six items) and two domains assessing feeling bothered by Emotions (four items) and Physical Problems (six items). As described for the adult version [54], scores are calculated for each of the QOLIBRI-KID/ADO scales, as well as a Total score for the entire questionnaire. Scores are expressed on a scale of 0–100. If the answer to the satisfaction questions (“How satisfied are you…?” and “How satisfied do you think your child is with...?”) was “not applicable” (N/A), the response was treated as a missing value. If the answer to a question about feeling bothered (“How bothered are you…?” and “How much do you think it bothers your child…?”) was N/A, it was assumed that the responder did not feel bothered by the issue, and the item was given a score of five.

The King’s Outcome Scale for Childhood Head Injury (KOSCHI) [55] clinically assesses functional recovery and disability burden after pTBI. The five categories of the KOSCHI are 1. death, 2. vegetative status, 3a/b. severe disability, 4a/b. moderate disability, and 5a/b. good recovery. 

Parents provided data on their own characteristics and those of their children/adolescents. The following child and adolescent socio-demographic and health data were collected from the parents: sex (male/female), presence of chronic diseases (yes/no), treatment for mental health disorders before TBI (yes/no). Parents also provided socio-demographic and health information about themselves: age, level of parental education (primary, secondary/high school, post-high school training, or university degree), and living arrangements (in a relationship or single parent). Finally, they completed the proxy version of the QOLIBRI-KID/ADO.

Each child and adolescent completed the QOLIBRI-KID/ADO in a face-to-face interview, either in person or online.

Clinicians or psychologists supplemented the data with details of the children’s (KID: 8–12 years) and adolescents’ (ADO: 13–17 years) age, time since injury, TBI severity (mild, moderate, or severe), and loss of consciousness (LOC), which was assessed using the GCS [53] or according to the International Classification of Diseases (ICD-10) diagnostic codes (S06.*) [56] in the medical records. If this information was missing, a clinical description of the TBI was aggregated from data on post-traumatic amnesia (PTA), need for ventilation and resuscitation, nausea/vomiting, post-traumatic epilepsy, presence of lesions (according to MRI/CT findings), the need for surgical intervention, and the injury severity score (ISS). Current post-injury functional recovery status was measured using the KOSCHI score [55] (good recovery, moderate disability, or severe disability).

### 2.3. Ethical Approval

The QOLIBRI-KID/ADO study was conducted in accordance with all relevant laws of Germany, including but not limited to the ICH Harmonized Tripartite Guideline for Good Clinical Practice (“ICH GCP”) and the World Medical Association’s Declaration of Helsinki (“Ethical Principles for Medical Research Involving Human Subjects”). The study received ethical clearance at each recruitment center and informed consent was obtained from all participants in line with German data protection laws (General Data Protection Regulation, DSGVO). The study was approved by the Ethics Committee of the University Medical Center Goettingen (application number: 19/4/18).

### 2.4. Data Analyses

Parent–child agreement was assessed for each QOLIBRI-KID/ADO item and scale, and for the Total score. Analyses were conducted for the total sample and for the two age groups. Continuous variables were summarized using means, standard deviations (SD), medians, and ranges. Categorical data were presented as frequencies (N) and percentages (%). The significance of the different tests was fixed at *p* ≤ 0.05. All score distributions were checked for normality; if there was evidence of non-normality or the frequencies for categories were low, non-parametric methods (e.g., Fisher’s exact test) were applied. Mean child/adolescent and parent scores with 95% confidence intervals (CIs) and the proportion of missing data were reported for each HRQoL scale and the Total score. Directional and absolute differences between child/adolescent and parent QOLIBRI-KID/ADO scores were summarized for each scale and for the Total scores.

QOLIBRI-KID/ADO scores were computed when no more than one-third (33%) of the items per scale was missing, and Total scores were computed only when scale scores were available. SAS software version 9.4 [57] was used. The PROC LCA procedure (http://methodology.psu.edu (accessed on 1 February 2022) [58]) was used to undertake the latent class analysis. For the PROC LCA procedure, only complete cases could be considered. 

#### 2.4.1. Internal Consistency

Internal consistency was assessed by means of McDonald’s omega (ω) and Cronbach’s alpha (α) coefficients for each QOLIBRI-KID/ADO scale, for the self-report and parental responses within each age group, and overall. Based on the results in similar pediatric populations, McDonald’s ω was considered satisfactory if it was at least 0.7, e.g., [59]. Cronbach’s α reliability coefficient was considered satisfactory if it was at least 0.7 for parent reports [60] and at least 0.6 for child/adolescent reports, e.g., [61]. The different cut-offs used for children and adults reflect the fact that Cronbach’s α cannot be considered a general measure of the scale or instrument itself, but only of its application to a particular sample [62]. It has been noted that in pediatric samples, Cronbach’s α is often found to be < 0.6 for single dimensions/subscales. Therefore, 0.6 is applied as a critical cut-off for the usefulness of these subscales in research and clinical work [63].

#### 2.4.2. Parent–Child Agreement for the QOLIBRI-KID/ADO

We used two approaches to assess parent–child agreement, each investigating different aspects of concordance. Firstly, the intraclass correlation coefficient (*ICC*) was calculated as an estimate of the magnitude of the association [63] between self- and parent-reported scores. The *ICC* considers rater bias, with a small bias indicating good agreement. *ICC* values range from 0 (no agreement) to 1 (perfect agreement). We used the following cut-offs in interpreting the scores: *ICCs* < 0.40 were considered poor, 0.40–0.60 fair, 0.60–0.80 good, and ≥ 0.80 excellent agreement [64]. The *ICCs* were calculated for each age group, for each scale, and for the Total score using a linear mixed repeated measures model. In addition, the model was adjusted for the type of parent completing the questionnaire to assess whether reporting by mothers or fathers had an impact on the agreement.

Secondly, Cohen’s Kappa (κ) [65] was calculated for each QOLIBRI-KID/ADO item and for each scale as an estimate of inter-rater observer agreement, taking into account the expected agreement by chance. In view of the ordinal nature of the item response scales, a weighted κ was used with linear weights assigning equal importance to the differences between any two categories within the response scale. κ ranges from −1 to 1; the following ranges describe the relative strength of agreement: poor < 0, slight (0–0.20), fair (0.21–0.40), moderate (0.41–0.60), substantial (0.61–0.80), (almost) perfect (0.81–1.00) [66]. A κ of 0 means there is no difference between the observers and chance alone [67]. We assumed that parent–child agreement would be better than expected by chance and therefore a one-tailed test is reported.

#### 2.4.3. Factors Associated with Parent–Child Agreement Concerning HRQoL

Factors associated with the agreement between the disease-specific HRQoL reported by children/adolescents and by their parents were investigated. The impact of child and adolescent characteristics on parent–child agreement (dependent variable) was analyzed using logistic regressions, including age (KID or ADO), sex (male or female), TBI severity (mild, moderate, or severe), KOSCHI score (5a/b, 3a/b, or 4a/b), presence of chronic diseases (no, yes), and presence of mental health disorder prior to TBI (no, yes). Children’s/adolescents’ and parents’ reports were defined as being in agreement (KID/ADO = Parent) if the absolute difference between their scores was less than or equal to half a standard deviation, e.g., [68] of the child’s scores for that age group. Two possible categories of disagreement were identified: First, the child’s reported score was higher than the parent’s score (KID/ADO > Parent) and, second, the child’s score was lower than the score given by their parent (KID/ADO < Parent). Due to the relatively small sample size, the two types of disagreement (KID/ADO < Parent and KID/ADO > Parent) categories were merged into one disagreement category when analyzing agreement. 

Differences in agreement were compared using chi-square tests with respect to the summarized characteristics of children and adolescents, households, and parents according to the membership in the agreement group. The estimated associations were analyzed using odds ratios (ORs; equality: OR = 1, higher chance for the numerator group: OR > 1, higher chance for the denominator group: OR < 1). Logistic regression models were constructed using each child and adolescent variable separately to model the effect on agreement. This approach was followed by multivariable logistic regression, adding all the factors to the model and undertaking a stepwise selection procedure. The level for a variable to enter and be retained in the model was set at 0.30. The models were run for each scale and the Total score over the entire study sample. 

A latent class analysis (LCA) approach was used to understand whether the children and adolescents in our sample were made up of certain subgroups based on specific characteristics. We investigated whether there were latent classes of children and adolescents based on their pre-TBI, TBI characteristics, parent education, and parent–child agreement, and, if so, what their nature and prevalence were. The following indicator variables were used in determining child and adolescent latent groups: age (coded as: 1 = KID, 2 = ADO); sex (1 = female, 2 = male); TBI severity (1 = mild, 2 = moderate/severe); KOSCHI (1 = 5a/b, 2 = 3a/b, 4a/b); presence of mental disorder prior to TBI (1 = No, 2 = Yes); presence of chronic disorder prior to TBI (1 = No, 2 = Yes); parental education (1 = University, 2 = Other than university); and agreement coded as a dummy variable KID/ADO < Parent (1 = No, 2 = Yes), and KID/ADO > Parent (1 = No, 2 = Yes). The baseline model was estimated to specify the number of latent classes from the indicator variables. Models with up to five classes were fitted, and the best-fitting model was selected. The criteria used to assess the best model were likelihood-ratio *G*^2^ statistic (*LRT G*^2^), Akaike’s Information Criterion (*AIC*) [69], the Bayesian Information Criterion (*BIC*) [70], and entropy [71]. A smaller *AIC* and *BIC* suggest a better fit. For entropy, values close to 1 [72] are ideal; entropies above 0.6 are considered acceptable [73]. The models were compared and the one with the lowest *LRT G*^2^, *AIC*, and *BIC*, and the highest entropy, which was in addition the clinically most meaningful, was selected.

## 3. Results

### 3.1. Study Sample

The pilot study included 300 parent–child dyads, 152 in the child group and 148 in the adolescent group. Overall, there was a higher proportion of males (59.7%) than females (40.0%). A high proportion of TBIs were mild (71.7%) compared with moderate (8.3%) or severe (20.0%). The majority of children and adolescents (89.7%) recovered well (KOSCHI score 5a/b). Forty-two percent of participants were interviewed four to ten years after their TBI. One quarter of the parents reported their child/adolescent having chronic diseases and 8.7% children and adolescents had received treatment for a mental disorder prior to their TBI. Statistically significant differences between the children and adolescent groups were found in functional recovery after TBI; a high proportion of those in the child group (94.7%) had good recovery (KOSCHI score 5a/b) compared with those in the adolescent group (84.5%). For details, see Table 1.

A higher proportion of mothers (79.7%) than fathers (18.7%) completed the socio-demographic and HRQoL questionnaires, and half of the proxies who completed the questionnaires attended university. A high proportion of proxies were living in a relationship (87.3%) rather than being single parents (10.0%). See Table 2 for details.

### 3.2. Descriptive Statistics of the QOLIBRI-KID/ADO Questionnaire

**Figure 1:**
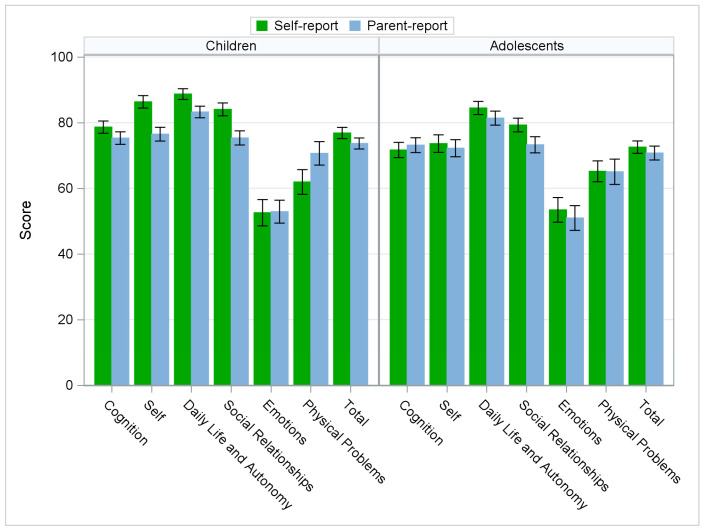
QOLIBRI-KID/ADO mean scores for parent and self-reports.

**Figure A1:**
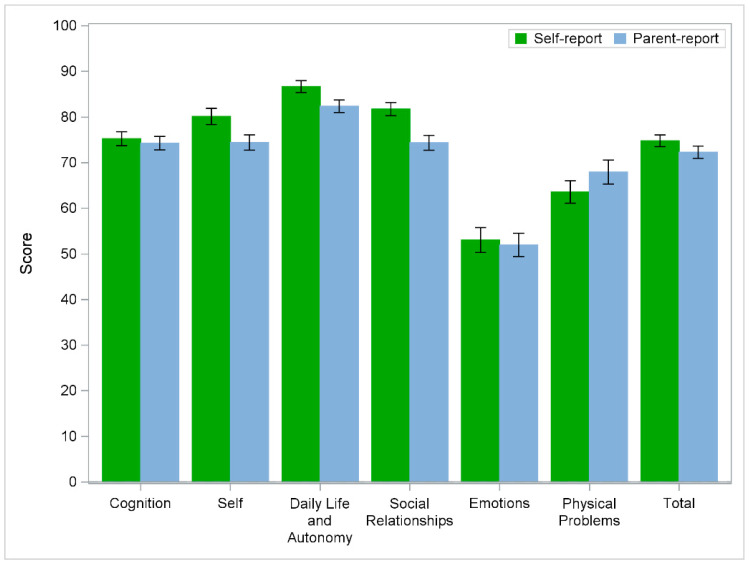
QOLIBRI-KID/ADO self- and parent-reported mean scores for the total sample.

Summaries of the QOLIBRI-KID/ADO mean scores by type of completion (self or parent) and by age group are shown in Table A1 (Appendix A) and summarized graphically in Figure 1. Mean scores for the total sample are presented in Figure A1, Appendix A.

Compared with parent ratings, children reported higher mean scores on all but the Physical Problems and Emotions scales. The largest directional mean differences between child and parent scores were seen for the scales Self (10 points), Social Relationships (8.7 points), and Physical Problems (−8.7 points). The largest absolute differences were found for the Emotions (23.9 points) and Physical Problems (22.8 points) scales. 

**Figure A2:**
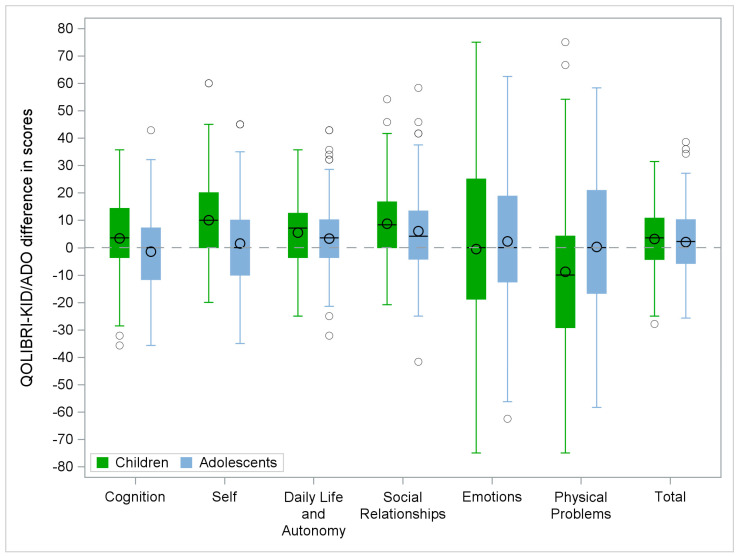
Differences in self- and parent reports in QOLIBRI-KID/ADO scores by age category.

In the adolescent group, the mean self-reported scores were higher than the parent-reported scores on all scales except the Cognition scale. The largest directional mean differences between adolescent and parent scores were observed on the Social Relationships scale (6.0 points) and the largest absolute difference was on the Emotions scale (21.7 points). The largest SDs in the difference were for the Emotions and Physical Problems scales (Figure A2, Appendix A).

Note: Mean scores and standard deviations (within 95% confidence intervals) are expressed on a scale of 0–100 by age group, with higher scores indicating higher HRQoL.

### 3.3. Data Analyses

#### 3.3.1. Internal Consistency

Cronbach’s α and McDonald’s ω for the scales and the total QOLIBRI-KID/ADO score by age group and overall are summarized in Table A2 (Appendix B). In the total sample, all α and ω values were above 0.70. In all scales except the Self scale, the reliability of the parent version was equal to or greater than the reliability of the self-report versions. 

In the child group, the reliability coefficients were above 0.60 for the Total score and all scales of the self-completed questionnaires. For the parent-completed questionnaires, α and ω were below 0.70 only for the Self scale (α = 0.69, ω = 0.66). For all scales except the Emotions scale, reliability values for the parent-completed questionnaires were equal to or greater than those for the child-completed questionnaires.

In the adolescent group, α and ω were above 0.60 for all self-completed scales and the total QOLIBRI-KID/ADO score. For all parent-completed scales and the Total score, α and ω were above 0.70. In this group, all values of α and ω were higher for the parent-completed questionnaires than those completed by adolescents for all scales and the Total score.

#### 3.3.2. Parent–Child Agreement of the QOLIBRI-KID/ADO

**Figure A3:**
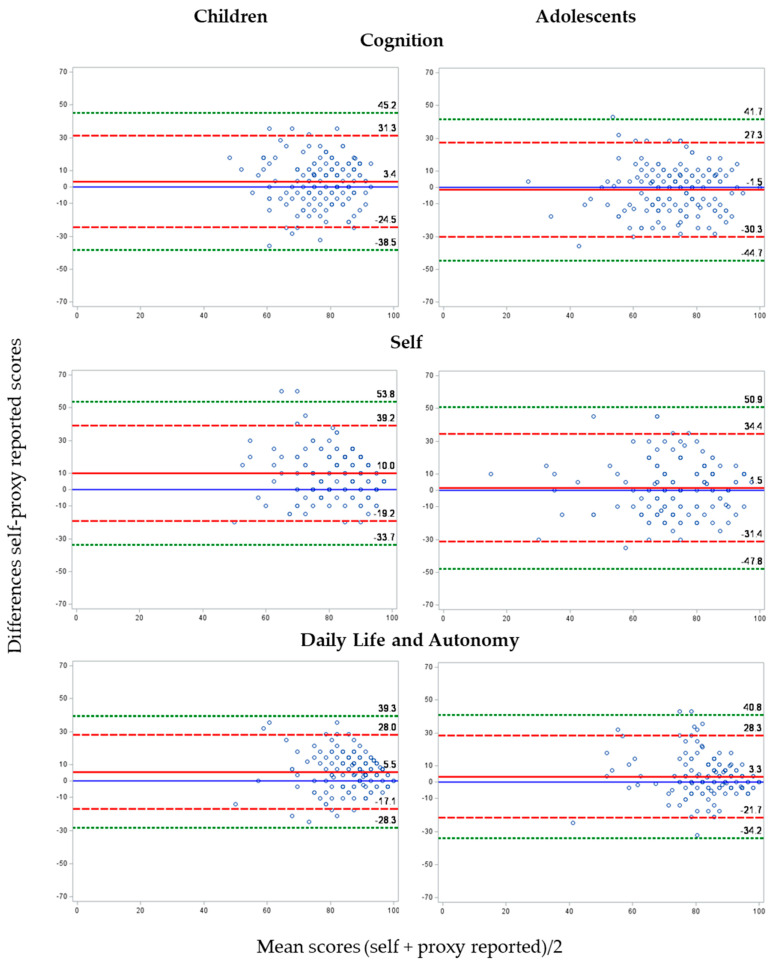

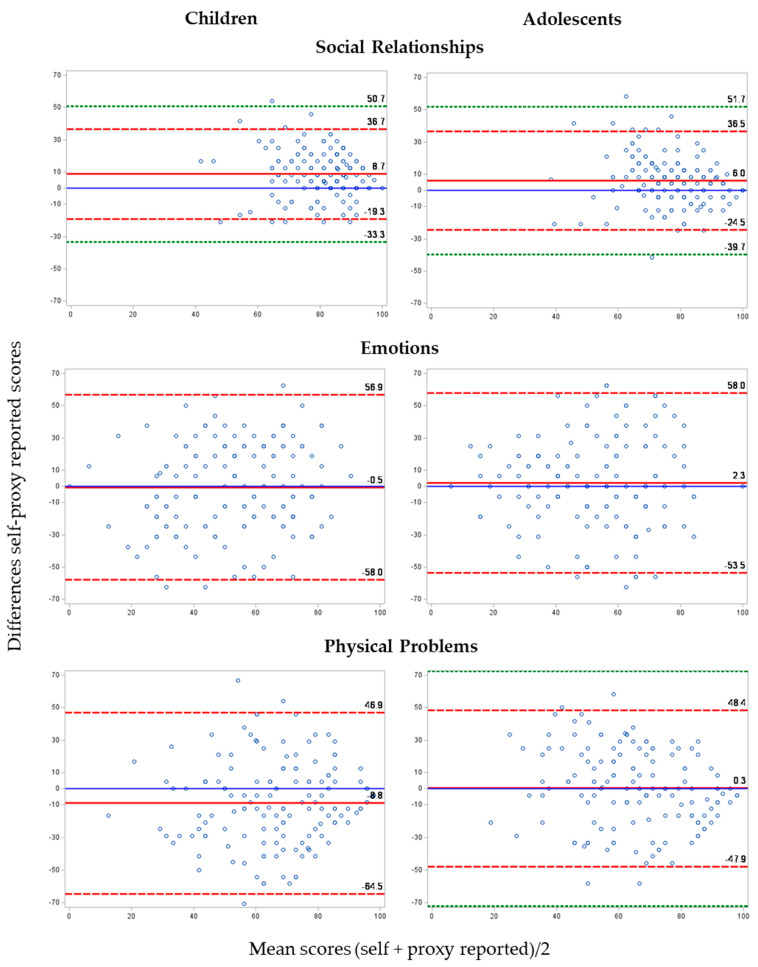

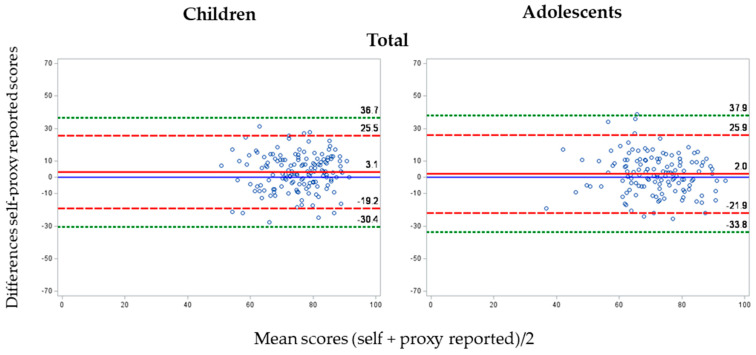
Bland–Altman plots for QOLIBRI-KID/ADO scales and Total scores by age groups.

Estimates from the simple linear mixed repeated measures models indicated moderate reliability (*ICC* ≥ 0.5) only on the Daily Life and Autonomy scales and for the Total scores in the adolescent group (Table 3). Poor concordance was found for all other scales. In the child group, reliability was poor (*ICC* < 0.5) for all scales and the Total score. Refining the model by adding type of parent (mother, father, or other) did not change these conclusions. We found no evidence of any statistically significant differences between different parent responders. Estimates of the *ICC* from the refined model were similar to those from the simple linear mixed model. Graphical summaries of agreements are presented in the Bland–Altman plots (Figure A3, Appendix B). 

In terms of the item-level inter-rater reliability, the relative strength of agreement measured by Cohen’s weighted κ was moderate (ranging from 0.41–0.60) in the adolescents’ group for only one item (“How satisfied are you with how you are able to concentrate at school?”). None of the agreement measures in the child group had moderate strength. The strength of agreement for the Total score was slight (0–0.20): 0.11 in the adolescent group and 0.09 in the child group. On the scale level, a fair (0.21–0.40) agreement was seen for the Cognition, Self, Daily Life and Autonomy, Social Relationships, and Physical Problems scales in the adolescent group. In the child group, fair agreement was seen for Cognition, Daily Life and Autonomy, Social Relationships, and Physical Problems scales (Table A3, Appendix B). 

#### 3.3.3. Factors Associated with Parent–Child Agreement Concerning HRQoL

The results of a simple bivariate logistic regression (Table A4, Appendix C) showed that only age was a significant predictor of parent–child agreement for the Cognition and Daily Life and Autonomy scales. The odds ratios for the children versus adolescent groups for agreement were less than one: 0.44 for agreement on Cognition (*p* = 0.001) and 0.50 for agreement on Daily Life and Autonomy (*p* = 0.004), suggesting that there are statistically significant differences between the age groups, and there is a lower probability of children agreeing with their parents than of an agreement between adolescents and their parents. For all other variables, e.g., TBI severity, in the bivariate logistic regression analysis, there was no evidence to suggest a significant association with parent–child agreement.

The results of the multivariable logistic regression (see Table 4) supported the findings from the bivariate analysis, with the addition of an association between the presence of chronic disease and agreement on the Emotions scale. Children and adolescents with chronic conditions were less likely to agree with their parents than children and adolescents without chronic conditions (*OR* = 0.52).

**Figure 2:**
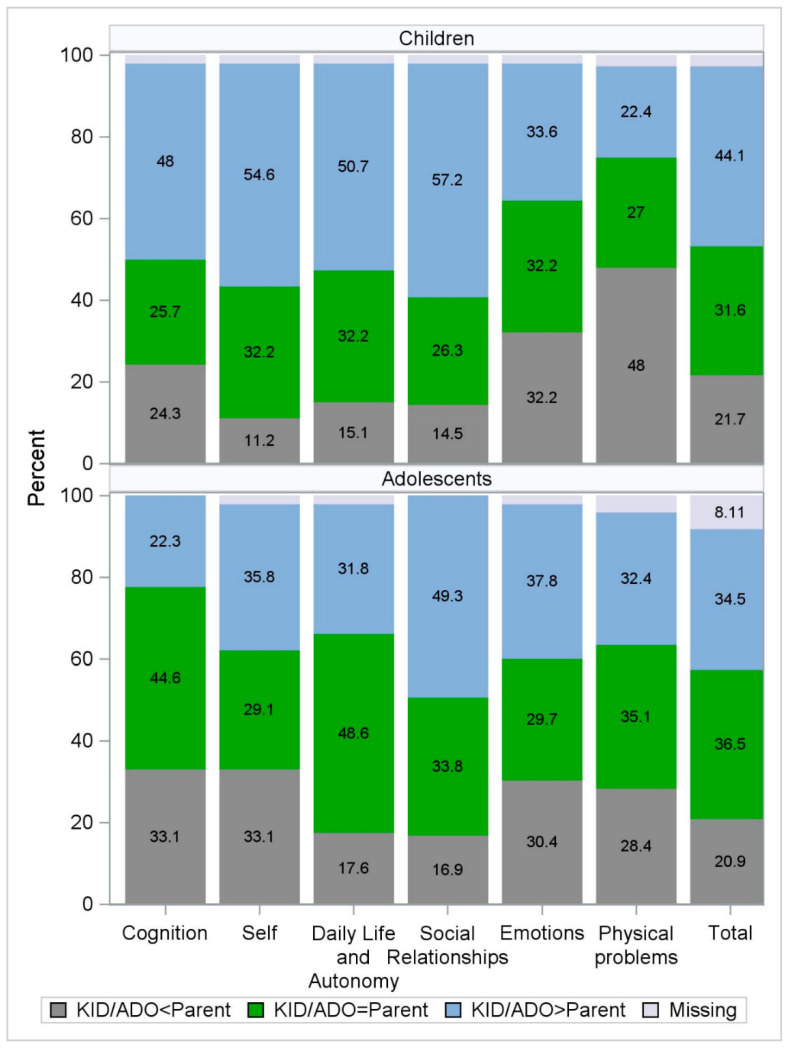
Agreement pattern between children, adolescents, and parents, proportion of dyads (in %) in each agreement category by age group and for each scale, and the Total score.

Parent–child agreement is summarized graphically in Figure 2. The threshold for agreement was different for each scale, and values (half *SD*) ranged from 5.1 to 12.5 points in the child group and between 5.7 and 11.5 points in the adolescent group. In the child group, the agreement rates were similar for all scales and ranged from 25.7% (Cognition) to 32.2% (Self, Daily Life and Autonomy, and Emotions). In the disagreement groups, a smaller proportion of children reported higher HRQoL than their parents on the Physical Problems scale compared to those reporting lower HRQoL than their parents (22.4% vs. 48.0%). In the adolescent group, the largest proportion of adolescents and parents agreed on the Daily Life and Autonomy scale reports (48.6%), followed by the Cognition scale (44.6%). The lowest proportion of the dyads agreed on the Emotions scale (29.7%). On all scales except the Cognition scale, a larger proportion of adolescents reported higher HRQoL than their parents compared to those who reported lower HRQoL than their parents.

Note: KID/ADO = Parent means agreement (absolute difference between child/adolescent and parent score is less than or equal to ½ SD of the child/adolescent values). KID/ADO < Parent means that parent overestimated the HRQoL of their child/adolescent. KID/ADO > Parent means that parent underestimated the HRQoL of their child/adolescent.

Sixteen dyads were excluded from the agreement summaries because at least one of the responders (parent or child/adolescent) had a missing QOLIBRI-KID/ADO Total score; Table 5.

There was evidence of a statistically significant but marginal difference in parent–child agreement (Table 5) for children/adolescents who received treatment for a mental disorder prior to injury (χ^2^ = 6.11; *p* = 0.047). These findings should therefore be interpreted with caution, also because of small effect sizes and the small sample size for children/adolescent receiving treatment for mental disorders (*n* = 25) compared to those without such a treatment (*n* = 256). The chi-square test in Table 5 suggests that the three agreement groups are not significantly different in terms of all the other variables (*p* > 0.05), including the hypothesized effect of TBI severity (*p* = 0.217).

To identify the factors influencing the three (dis-)agreement groups using the QOLIBRI-KID/ADO Total score, the LCA was performed for two to five classes with eight variables. A combination of the values of *G*^2^ statistic, *AIC*, *BIC*, entropy, and percentage of seeds fitting the model suggested that the three-class model was the best of four models (see Table 6) according to the criteria mentioned in the Section 2 (lowest LRT *G*^2^, AIC, and BIC, and the highest entropy).

**Figure 3:**
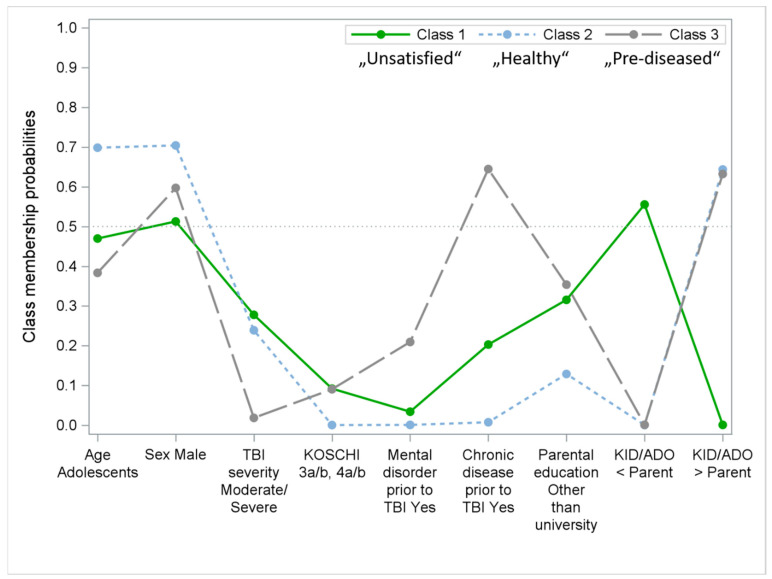
Latent profiles of child/adolescent characteristics of the three agreement classes (based on QOLIBRI-KID/ADO Total score).

Furthermore, this model is the clinically most plausible option, since the three groups are distinct in terms of their characteristics labeled “Unsatisfied”, “Healthy”, and “Pre-diseased”. The item response probabilities for each indicator variable for each latent class are presented graphically in Figure 3.

Class 1, labeled as “Unsatisfied”, was the largest class (47.6%). Participants in the “Unsatisfied” class reported lower HRQoL scores than their parents and were more likely to have had a more severe TBI and more chronic diseases. In class 2 (20.9%), labeled as “Healthy”, children and adolescents had higher HRQoL values than their parents, and there was an overrepresentation of male adolescents after mild TBI without any mental or chronic health problems. Class 3 (31.4%), “Pre-diseased”, consisted of children and adolescents after a rather mild TBI who had higher HRQoL scores than their parents, and the class is characterized by an overrepresentation of the presence of chronic diseases and a tendency towards having had more mental health problems prior to TBI.

## 4. Discussion

In this study, TBI-specific HRQoL was assessed for the first time in children/adolescents and their parents using the newly developed six-dimensional TBI-specific instrument, the QOLIBRI-KID/ADO. The agreement between pediatric self-reports and parental reports was analyzed. As there is a paucity of research on parent–child agreement on HRQoL after pTBI, our study contributes to the understanding of the convergence of the child/adolescent and the parental perspectives in this area. Factors that might potentially influence this agreement were explored. There is a consensus that parental reporting alone is not sufficient to describe HRQoL in children and adolescents in general, e.g., [37], and after pTBI in particular, e.g., [20]. The combination of child/adolescent and parental perspectives may provide a broad and comprehensive picture of children’s and adolescents’ HRQoL in order to improve pediatric health care after TBI [74]. It is, however, unclear which type of assessment should be used, and when, and whether they should be combined, e.g., [11].

Because of the disparities between self- and proxy reports, parental reports should only be used as a surrogate when the child is too young or has difficulty understanding the constructs being assessed [23,31], cognitive deficits (e.g., [23]), or a possible lack of awareness [48]. There is a paucity of literature exploring the reasons or factors that lead to such disparities between self- and proxy reports after pTBI. We therefore analyzed possible factors that might explain differences in agreement, such as age, sex, severity, functional recovery, and chronic and mental problems prior to pTBI.

The results of the present study indicate good psychometric properties of the QOLIBRI-KID/ADO scales for self-reporting and parental reporting of HRQoL in children and adolescents.

The distribution of sex and severity corresponds to the epidemiological frequencies determined in Germany [75], with more males affected (59.3%) than females (40.0%), and more mild TBI (71.7%) due to the small number of participants after moderate (8.3%) and severe (20.0%) TBI. The majority of the children/adolescents (89.7%) and their parents (82.3%) were quite satisfied (corresponding to the transformed QOLIBRI-KID/ADO scores ≥ 80), as expressed in the QOLIBRI-KID/ADO Total score; they also reported not being bothered too much by physical or emotional problems. These findings may be explained by the relatively long time since injury in the current study sample; 42.0% of the TBIs had occurred four to ten years ago. Several studies have found that HRQoL post-TBI improves over time, e.g., [76], especially during the first year [77]. The higher HRQoL could also be explained by the higher proportion of children/adolescents with good functional recovery [78] (in our study 89.7%) or after mild TBI, e.g., [3]. Other reasons for high HRQoL scores could be the absence of chronic diseases [79] (in our study 73.0%) or the absence of any treatment for mental disorders prior to TBI, e.g., [80] (in our study 89.0%).

Irrespective of TBI severity and age, children/adolescents and their parents reported the highest scores for the Daily Life and Autonomy scale and the lowest for the Emotions and Physical Problems scales. The high scores for the Daily Life and Autonomy scale are in contrast to findings for children and adolescents with chronic diseases and mental health issues [81]. Perceived autonomy in adolescence has been found to be positively related to HRQoL, e.g., [82]. According to self-determination theory, autonomy is one of the basic psychological needs of adolescents (and adults) [83], which may explain the high satisfaction scores in our sample.

Lower scores for physical and emotional HRQoL after TBI compared to other scales are consistent with findings from other studies on long-term consequences after pTBI [84,85,86,87,88,89]. The low scores on the Emotions scale support the notion that children and adolescents are particularly psychologically and emotionally distressed after TBI, even years after the injury. It has been shown that children and adolescents after TBI are at increased risk of developing mental and psychological symptoms, e.g., [90]. However, in our study we found that the bothered scales displayed fewer ceiling effects and more variance in responses compared with the satisfaction scales.

Overall, the conclusions from our study concerning parent–child agreement are in line with existing literature findings that the concordance between parents and children after TBI is rather poor and parents tend to underestimate their children’s HRQoL, e.g., [3,15]. There was one exception: on average, parents rated children’s HRQoL significantly higher on the Physical Problems scale than the children themselves. In contrast, previous research suggests that more internalized HRQoL dimensions (e.g., emotions) may be perceived and rated better by children themselves and lower by parents compared with more observable functioning (e.g., physical generic HRQoL), e.g., [24]. In the current study, opposite results were found, which might be due to the higher proportion of children after mild TBI with no physical complaints identified by parents. However, the same complaints were perceived differently by children. A response bias towards negatively or positively worded items could be part of the explanation, e.g., [91].

Taking the *ICC* into consideration, we found poor to moderate concordance in parent–child agreement on HRQoL for all scales and the Total score. However, the *ICC* depends strongly on the variance in pre-injury and injury-related characteristics of the population being assessed. Consequently, despite similar levels of agreement, the *ICC* will be lower in a more homogeneous population [92]. In summary, while there were small mean differences between self- and parent-reported scores in some of the adolescents’ scores (Cognition, Self, Emotions, and Physical Problems scales) and in the child group (Emotions scale), other findings showed a weak reliability (indicated by a small *ICC*) and wide agreement intervals in Bland–Altman plots. The findings point to a large variation in the differences in parent–child scores in our sample, indicating a lack of robust parent–child agreement. This is consistent with the findings in the pediatric literature assessing HRQoL in healthy (e.g., [25]) or ill (e.g., [93]) children and adolescents, including those after TBI [28,35]. These studies also report poor to moderate concordance between parents and children.

Several reasons could explain the higher self-rated HRQoL compared with the parent-rated HRQoL in children and adolescents after TBI. It is possible that children/adolescents and parents interpret situations differently due to different realities and perspectives [94]. Parents may have specific concerns when caring for their child after TBI [95]. The presence of psychosocial stressors could also negatively affect parent–child communication and the extent to which a parent is able to reflect on the child’s HRQoL [96].

The most important predictor to consider when analyzing child and parent HRQoL data is the child’s age: stronger agreement between self- and parent-reported scores was observed in the adolescent group. Based on the review of Bland–Altman plots, *ICCs*, and multivariable analysis model outcomes, the best agreement between self and parent was found in the adolescent group on the Cognition and Self scales, and for the Total scores. In the child group, statistically significant differences between self- and parent reports were found on all scales except the Emotions scale. However, both the unadjusted and adjusted *ICCs* for the Emotions scale were low (below 0.24), suggesting poor agreement between child and parent scores for younger children. In contrast to our study, some other studies have reported that agreement decreased with age in healthy children and adolescents, e.g., [25], as well as in children and adolescents after TBI, e.g., [46]. Due to the developmental level of the younger children, their perception of the own HRQoL may differ from that of their parents more than that of adolescents, who may be cognitively more mature and therefore have more similar perceptions to their parents.

The results of the current study exploring potential factors influencing agreement and disagreement by bivariate and multivariable logistic regression analyses confirm the importance of the child’s and adolescent’s age. However, neither logistic regression nor latent class analyses indicated that TBI severity, sex, functional recovery, or child and adolescent mental health problems were predictive of parent–child agreement. Other studies have reported inconsistent results regarding the effect of TBI severity on concordance, ranging from no effect, e.g., [26] to an effect with lower or even a greater concordance [28] with higher TBI severity. In the current study, TBI severity was not found to have any influence on parent–child agreement. Robust subgroup analyses by agreement groups were not possible due to the low frequencies in the moderate/severe severity groups (*n* = 17, 25, 41, respectively). Therefore, we recommend that the effect of TBI severity is investigated in further studies using different samples. Additionally, one should be cautious in the interpretation, since most of our participants had experienced a mild TBI.

To the best of our knowledge, only one study examining the impact of sex reported that children belonging to the female sex predicted higher parent scores in psychosocial health [29], and the results of the current study do not support this finding. The finding in our study that children after TBI are less likely to agree with their parents on the Emotions scale than children without any chronic conditions should not be over-interpreted. Other factors, such as parental distress [97], parental practices [98], or family functioning, e.g., [99], may have an impact on parent–child agreement. Further studies are needed to support our findings or to broaden the view of the factors that influence parent–child agreement on HRQoL.

### 4.1. Strengths and Limitations

The current study has a number of strengths. Our sample provides valuable data on children/adolescents and their parents following pTBI. Collecting such data in this population is challenging because of the high drop-out rate (80–90%). This is maybe due to parental fears of re-traumatizing their children by the study participation or by the fact that parents did not see the benefit of participating when their children were no longer symptomatic. Therefore, to the best of our knowledge, this study is one of the first to examine factors contributing to differences in parent–child agreement on HRQoL after pTBI using multiple statistical methods. The current findings are a promising start towards a better understanding of different perspectives of parent–child agreement, family risks, and protective factors for improving HRQoL in children and adolescents after TBI.

An important limitation is the cross-sectional design, which is due to the aim of the main study, i.e., to develop a new TBI-specific HRQoL questionnaire. It may be seen as an important limitation of our study that the data were assessed at different time points after TBI, which could differ from three months up to ten years post-injury. Because the number of individuals after TBI less than one year prior to study enrollment was very small (*n* = 16 in the children and *n* = 10 in the adolescent group), no other analyses comparing early recovery (up to one year post TBI) with long-term recovery (one year and more) could be undertaken. This limits our analyses and conclusions, and we strongly recommend that future studies consider time since injury. Furthermore, in our study, predominantly individuals after mild TBI who had no cognitive or awareness problems participated. Studying the impact of TBI in children and adolescents after moderate to severe TBI, with a moderate recovery, comorbidities, formal assessment of self-awareness, etc., should be intensified in prospective, longitudinal studies. Parent and child/adolescent views should be further investigated to better understand the relationship between the two perspectives and to derive relevant information for the evaluation of therapy and rehabilitation after TBI.

### 4.2. Recommendations

Given that there may not be full parent–child concordance in reporting disease-specific HRQoL after pTBI, future research needs to further clarify and examine these differences. Using a longitudinal study design would allow the following aspects to be investigated: self- and parent perspectives in parallel in order to explore children’s and adolescent’s HRQoL over time after pTBI; the effect of improvement or deterioration in the recovery process; the presence of chronic diseases or mental health problems in children and adolescents; and the impact on parental reports. Furthermore, the identification of latent agreement classes indicates the need for a separate investigation of the groups found. Before carrying out this investigation, the latent classes should be validated using another sample after pTBI with more balanced severity groups and potential risk factors. A key implication of this study is that children and adolescents after TBI should primarily be evaluated by means of self-reporting, especially because self-reports after pTBI are underrepresented in clinical and research work [17,19]. Only when children cannot respond themselves should parental reports be used as a surrogate for their perspective. Clinicians and researchers may then use multi-informant assessments (at least self- and parent reports) of HRQoL to personalize diagnosis and treatment after pTBI.

## 5. Conclusions

The current study underlines the differences between self- and parent reports on disease-specific HRQoL in children and adolescents after TBI. The age of the children was found to be the most important factor explaining these differences. The agreement between self- and parent reports displayed poor to moderate concordance, with parents tending to report lower HRQoL in their children. Our results emphasize the importance of evaluating self-reported HRQoL in children and adolescents after TBI and of using proxy reports only in cases where the children and adolescents cannot answer for themselves. Furthermore, observer-ratings should be used when a comparison of both perspectives is the clinical or research question, for example, in order to enhance the therapeutic process regarding different views of parents and children on daily life aspects.

## Figures and Tables

**Table 1:** Socio-demographic and clinical descriptive data of children and adolescents.

		Children (*n* = 152)	Adolescents (*n* = 148)	(Test Statistic, *df*) *p*-Value *	Total (*n* = 300)
Age (years)	Mean (SD)	10.6 (1.40)	15.2 (1.47)	N/A	12.9 (2.72)
Sex				(0.50, 1) 0.479	
Female	N (%)	58 (38.2)	62 (41.9)		120 (40.0)
Male		94 (61.8)	85 (57.4)		179 (59.7)
Missing		0 (0.0)	1 (0.7)		1 (0.3)
TBI severity				(1.95, 2) 0.377	
Mild	N (%)	106 (69.7)	109 (73.6)		215 (71.7)
Moderate		16 (10.5)	9 (6.1)		25 (8.3)
Severe		30 (19.7)	30 (20.3)		60 (20.0)
KOSCHI				(8.55, 1) **0.004**	
3a/b, 4a/b	N (%)	8 (5.3)	23 (15.5)		31 (10.3)
5a/b		144 (94.7)	125 (84.5)		269 (89.7)
Time since injury (years)				(2.16, 3) 0.541	
<1	N (%)	16 (10.5)	10 (6.8)		26 (8.7)
1 to <2		48 (31.6)	43 (29.1)		91 (30.3)
2 to <4		29 (19.1)	27 (18.2)		56 (18.7)
4 to 10		59 (38.8)	67 (45.3)		126 (42.0)
Missing		0 (0.0)	1 (0.7)		1 (0.3)
Chronic diseases				(0.55, 1) 0.457	
No	N (%)	116 (76.3)	103 (69.6)		219 (73.0)
Yes		36 (23.7)	39 (26.4)		75 (25.0)
Missing		0 (0.0)	6 (4.1)		6 (2.0)
Treatment for mental disorder before the TBI				(2.64, 1) 0.104	
No	N (%)	137 (90.1)	130 (87.8)		267 (89.0)
Yes		9 (5.9)	17 (11.5)		26 (8.7)
Missing		6 (3.9)	1 (0.7)		7 (2.3)

Note: *n* = number analyzed, * Test of differences between age groups: Categorical variables: χ^2^ test.

**Table 2:** Socio-demographic data of parents.

Parent Characteristics		Children (*n* = 152)	Adolescents (*n* = 148)	(Test Statistic, *df*) *p*-Value	Total (*n* = 300)
Questionnaire completed by				(0.32, 1) 0.572	
Mother	N (%)	118 (77.6)	121 (81.8)		239 (79.7)
Father		30 (19.7)	26 (17.6)		56 (18.7)
Other person		4 (2.6)	1 (0.7)		5 (1.7)
Parent age (years)	Mean (SD) Missing	44.6 (5.15) 26	48.0 (5.64) 41	(4.74, 231) **<0.001**	46.2 (5.63) 67
Education of parent completing the questionnaire				(Fisher’s exact test) 0.218	
Primary school	N (%)	1 (0.7)	0 (0.0)		1 (0.3)
Secondary/high school		36 (23.7)	42 (28.4)		78 (26.0)
Post-high school training		21 (13.8)	29 (19.6)		50 (16.7)
University		82 (53.9)	68 (45.9)		150 (50.0)
Missing		12 (7.9)	9 (6.1)		21 (7.0)
Parent living in a partnership				(2.26, 1) 0.133	
Single parent	N (%)	11 (7.2)	19 (12.8)		30 (10.0)
In a relationship		134 (88.2)	128 (86.5)		262 (87.3)
Missing		7 (4.6)	1 (0.7)		8 (2.7)

Note: *n* = number analyzed, *SD* = standard deviation, *df* = degrees of freedom, Analyses of differences between age groups: Continuous variables with two-sample *t*-test, categorical variables with χ^2^ test (or Fisher exact test, if *n* < 5).

**Table 3:** Relationship between child and adolescent self-rated and parent-rated QOLIBRI-KID/ADO scales.

QOLIBRI-KID/ADO Scales and Total Score for Both Age Groups	Directional Differences (Child/Adolescent—Parent)				Mixed Linear Model ^2^				Type of Parent (Mother or Father *)		Absolute Differences |Child/Adolescent—Parent| Mean (SD)
	Mean (SD)	Missing	*p*-Value ^1^ Paired *t*-Test	*p*-Value Repeated Measures Model	Un-adjusted *ICC*	Mean Estimate (*SE*)	*p*-Value	Ad-justed *ICC*	*p*-Value ^3^ Repeated Measures	*ICC* Adjusted for Type of Parent	
Children (*n* = 152)											
Cognition	3.4 (13.95)	3	0.004	0.004	0.291	3.5 (1.10)	0.003	0.276	0.907	0.290	11.5 (8.49)
Self	10 (14.58)	3	<0.001	<0.001	0.308	10.2 (1.21)	<0.001	0.304	0.261	0.303	13. 8 (11.08)
Daily Life and Autonomy	5.5 (11.27)	3	<0.001	<0.001	0.429	5.7 (0.93)	<0.001	0.393	0.571	0.426	10.0 (7.57)
Social Relationships	8.7 (14.00)	3	<0.001	<0.001	0.401	8.8 (1.15)	<0.001	0.400	0.376	0.395	13.0 (10.11)
Emotions	−0.5 (28.74)	3	0.822	0.872	0.239	0.2 (2.34)	0.937	0.255	0.342	0.233	23.9 (15.87)
Physical Problems	−8.8 (27.86)	4	<0.001	<0.001	0.249	−8.7 (2.33)	<0.001	0.232	0.206	0.241	22.8 (18.20)
Total Score	3.1 (11.18)	4	<0.001	<0.001	0.436	3.4 (0.93)	<0.001	0.409	0.881	0.435	9.3 (6.89)
Adolescents (*n* = 148)											
Cognition	−1.5 (14.39)	0	0.214	0.211	0.478	−1.5 (1.22)	0.218	0.420	0.353	0.473	11.5 (8.72)
Self	1.5 (16.45)	3	0.271	0.285	0.483	1.1 (1.39)	0.419	0.438	0.753	0.482	13.2 (9.85)
Daily Life and Autonomy	3.3 (12.49)	3	0.002	0.003	0.509	2.8 (1.07)	0.010	0.471	0.701	0.506	9.1 (9.17)
Social Relationships	6.0 (15.24)	0	<0.001	<0.001	0.412	6.0 (1.31)	<0.001	0.363	0.604	0.409	12.3 (10.77)
Emotions	2.3 (27.87)	3	0.325	0.293	0.269	2.4 (2.42)	0.332	0.227	0.274	0.261	21.7 (17.53)
Physical Problems	0.3 (24.10)	6	0.897	0.926	0.368	0.3 (2.07)	0.883	0.303	0.475	0.364	19.2 (14.46)
Total Score	2.0 (11.96)	12	0.050	0.060	0.508	1.8 (1.05)	0.093	0.426	0.986	0.508	9.5 (7.46)

Note: Correlation ≤ 0.2 (very weak correlation), 0.2 ≤ 0.4 (weak correlation), 0.4 ≤ 0.6 (moderate correlation). ^1^ *p*-value: comparison between child/adolescent and parent. ^2^ Mixed repeated linear model adjusted for sex (female/male), TBI severity (moderate or severe/mild), KOSCHI (5a/b vs. 3a/b or 4a/b), Chronic disease (yes/no), pre-TBI mental disorder (yes/no). Output is the QOLIBRI-KID/ADO score by child, adolescent and by parent (repeated measures). ^3^ *p*-value: comparison between responses of mother or father. * Other person was excluded here as type of parent for statistical reasons, because this group was too small.

**Table 4:** Multivariable logistic regression of child/adolescent characteristics for parent–child agreement.

Variable in the Model	QOLIBRI-KID/ADO Scales						Total Score
*p*-Value Adjusted *OR* (95% *CI*)	Cognition	Self	Daily Life and Autonomy	Social Relationships	Emotions	Physical Problems	
Number of dyads in the model (%)	286 (95.3)	283 (94.3)	283 (94.3)	286 (95.3)	283 (94.3)	280 (93.3)	274 (91.3)
Age:	0.001		0.002			0.176	0.136
Children vs. Adolescents	0.41 (0.25, 0.68)		0.46 (0.29, 0.75)			0.71 (0.43, 1.17)	0.68 (0.41, 1.13)
Sex:			0.274		0.054	0.216	
Female vs. Male			0.76 (0.46, 1.24)		1.66 (0.99, 2.77)	1.38 (0.83, 2.30)	
TBI severity:	0.277			0.245		0.255	
Moderate or Severe vs. Mild	1.36 (0.78, 2.36)			0.71 (0.39, 1.27)		0.71 (0.40, 1.28)	
KOSCHI:							0.139
3a,b, 4a,b vs. 5a,b							0.50 (0.20, 1.25)
Chronic disease:					0.044		
Yes vs. No					0.52 (0.27, 0.98)		
Pre-TBI mental disorder:	0.192	0.140					
Yes vs. No	0.52 (0.19, 1.39)	0.43 (0.14, 1.31)					

Note: Modeling probability: Agreement = Yes. Variables in the model: Age, Sex, TBI severity, KOSCHI, Presence of chronic disease, Presence of pre-TBI mental disorder. Stepwise regression (all variables were initially in the model). Second category is the reference category. In bold, *p* < 0.05. Only variables fulfilling the entering (0.30) and retaining (0.30) criteria are presented (see Section 2). Final multivariable logistic regression models explaining agreement versus disagreement for each scale and Total. *OR* = odds ratio. *CI* = confidence interval.

**Table 5:** Sample characteristics by agreement between child, adolescent, and parent for Total QOLIBRI-KID/ADO score.

	KID/ADO < Parent (*n* = 64)	KID/ADO = Parent (*n* = 102)	KID/ADO > Parent (*n* = 118)	Total (*n* = 284)	(*χ^2^*, *df*) *p*-Value
Age category					(2.08, 2) 0.353
Children	33 (51.6%)	48 (47.1%)	67 (56.8%)	148 (52.1%)	
Adolescents	31 (48.4%)	54 (52.9%)	51 (43.2%)	136 (47.9%)	
Child sex					(5.38, 2) 0.068
Female	34 (53.1%)	38 (37.3%)	43 (36.4%)	115 (40.5%)	
Male	30 (46.9%)	63 (61.8%)	75 (63.6%)	168 (59.2%)	
Missing	0 (0.0%)	1 (1.0%)	0 (0.0%)	1 (0.4%)	
TBI severity					(3.05, 2) 0.217
Mild	47 (73.4%)	77 (75.5%)	77 (65.3%)	201 (70.8%)	
Moderate or severe	17 (26.6%)	25 (24.5%)	41 (34.7%)	83 (29.2%)	
KOSCHI					(3.42, 2) 0.181
3a/b, 4a/b	6 (9.4%)	7 (6.9%)	17 (14.4%)	30 (10.6%)	
5a/b	58 (90.6%)	95 (93.1%)	101 (85.6%)	254 (89.4%)	
Chronic diseases					(1.48, 2) 0.477
No	50 (78.1%)	77 (75.5%)	81 (68.6%)	208 (73.2%)	
Yes	14 (21.9%)	23 (22.5%)	33 (28.0%)	70 (24.6%)	
Missing	0 (0.0%)	2 (2.0%)	4 (3.4%)	6 (2.1%)	
Before the TBI, the child/adolescent had treatment for mental disorder			(6.11, 2) 0.047		
No	60 (93.8%)	94 (92.2%)	102 (86.4%)	256 (90.1%)	
Yes	2 (3.1%)	7 (6.9%)	16 (13.6%)	25 (8.8%)	
Missing	2 (3.1%)	1 (1.0%)	0 (0.0%)	3 (1.1%)	
Education (both parents)			(2.06, 2) 0.356		
University	43 (67.2%)	68 (66.7%)	71 (60.2%)	182 (64.1%)	
Other than university	20 (31.3%)	28 (27.5%)	44 (37.3%)	92 (32.4%)	
Missing	1 (1.6%)	6 (5.9%)	3 (2.5%)	10 (3.5%)	
Living in a partnership				(0.34, 2) 0.844	
Single parent	8 (12.5%)	10 (9.8%)	12 (10.2%)	30 (10.6%)	
In relationship	55 (85.9%)	90 (88.2%)	105 (89.0%)	250 (88.0%)	
Missing	1 (1.6%)	2 (2.0%)	1 (0.8%)	4 (1.4%)	
Questionnaire completed by				(5.06, 2) 0.080	
Mother	47 (73.4%)	89 (87.3%)	95 (80.5%)	231 (81.3%)	
Father	16 (25.0%)	12 (11.8%)	23 (19.5%)	51 (18.0%)	
Missing	1 (1.6%)	1 (1.0%)	0 (0.0%)	2 (0.7%)	

Note: Sixteen participants could not be included in the analysis as one or the other responder was missing a Total score (three for adolescents and thirteen for parents (four in the children and nine in the adolescent groups)).

**Table 6:** Latent classes of parent–child agreement: comparison of baseline models.

Number of Classes	df	LRT *G*^2^	AIC	BIC	Entropy	% of Seeds Associated with Best Fitted Model
2	492	294.0	332.0	401.3	0.70	65.0
**3**	**482**	**252.4**	**310.4**	**416.2**	**0.68**	**92.3**
4	472	226.2	304.2	446.5	0.69	18.0
5	462	204.7	302.7	481.5	0.74	13.3

Note: In bold—the selected model. LRT *G*^2^ = Likelihood ratio *G*^2^ test; AIC = Akaike’s Information Criterion; BIC = Bayesian Information Criterion; df = degrees of freedom.

## Data Availability

The data presented in this study are available on request from the corresponding authors. For reasons of data protection, the data are not publicly available.

## Appendix A

**Table A1:** QOLIBRI-KID/ADO self- and parent-reported scores split by age group.

	Self-Report		Parent Report	
	Mean (SD)	Missing	Mean (SD)	Missing
Children (*n* = 152)				
Cognition	78.6 (11.64)	0	75.3 (11.77)	3
Self	86.3 (11.83)	0	76.5 (12.94)	3
Daily Life and Autonomy	88.7 (10.20)	0	83.3 (10.89)	3
Social Relationships	84.0 (12.24)	0	75.4 (13.29)	3
Emotions	52.6 (25.02)	0	52.9 (21.56)	3
Physical Problems	61.9 (23.47)	0	70.7 (22.00)	4
Total	76.9 (10.71)	0	73.7 (10.33)	4
Adolescents (*n* = 148)				
Cognition	71.7 (14.29)	0	73.1 (13.88)	0
Self	73.6 (16.41)	1	72.2 (15.91)	2
Daily Life and Autonomy	84.5 (12.24)	2	81.4 (13.09)	1
Social Relationships	79.3 (12.85)	0	73.3 (15.17)	0
Emotions	53.4 (23.08)	0	51.0 (22.94)	3
Physical Problems	65.2 (19.58)	0	65.0 (23.25)	6
Total	72.6 (11.46)	3	70.8 (12.61)	9

Note: *n* = absolute frequency, SD = standard deviation.

Note: Mean scores and standard deviations are expressed on a 0–100 scale by age group with higher scores indicating higher HRQoL.

## Appendix B

**Table A2:** Cronbach’s α and McDonald’s ω for QOLIBRI-KID/ADO—agreement at the scale level by age category, response type, and Total score.

QOLIBRI-KID/ADO Scale	Children and Adolescents			Parents		
(*n* Items)	*n*	α	ω	*n*	A	ω
Cognition (7)	293	0.70	0.71	290	0.76	0.75
Self (4)	294	0.78	0.80	284	0.75	0.75
Daily Life and Autonomy (7)	284	0.70	0.71	276	0.74	0.75
Social Relationships (6)	296	0.73	0.72	283	0.78	0.77
Emotions (4)	295	0.72	0.72	289	0.72	0.72
Physical Problems (6)	288	0.73	0.73	270	0.77	0.76
Total (35)	266	0.89	0.88	235	0.91	0.90
	**Children**			**Parents**		
	*n*	α	ω	*n*	A	ω
Cognition (7)	152	0.64	0.63	146	0.71	0.73
Self (4)	151	0.68	0.68	145	0.69	0.66
Daily Life and Autonomy (7)	148	0.66	0.65	140	0.71	0.70
Social Relationships (6)	152	0.75	0.75	141	0.76	0.75
Emotions (4)	150	0.74	0.74	146	0.70	0.70
Physical Problems (6)	146	0.77	0.77	136	0.78	0.77
Total (35)	140	0.88	0.87	119	0.88	0.87
	**Adolescents**			**Parents**		
	*n*	α	ω	*n*	A	ω
Cognition (7)	141	0.72	0.73	144	0.78	0.78
Self (4)	143	0.78	0.79	139	0.78	0.79
Daily Life and Autonomy (7)	136	0.73	0.74	136	0.77	0.78
Social Relationships (6)	144	0.68	0.68	142	0.79	0.78
Emotions (4)	145	0.70	0.71	143	0.73	0.73
Physical Problems (6)	142	0.68	0.65	134	0.76	0.75
Total (35)	126	0.89	0.88	116	0.92	0.91

Note: *n* = absolute frequencies, α = Cronbach’s alpha, ω = McDonald’s omega.

### Bland–Altman Plots

Agreement between child and parent scores was visualized using Bland–Altman plots [100]. For each parent–child dyad (Figure A3, Appendix B), the mean of the parent and child score was plotted on the *x*-axis against the mean difference between the child and parent score on the *y*-axis. This approach provides information on comparability of the dyads by examining the difference between child and parent scores, the magnitude (mean of the child and parent score), and information on any potential patterns and outliers. There appears to be a larger bias (difference between the means in self- and parent scoring) in the Cognition, Self, Daily Life and Autonomy, Social Relationships, and Physical Problems scales, and Total scores, in the child group compared with the adolescent group, with self-rating scores being higher on average than the parental scores. Concerning the Physical Problems scale, children reported lower scores compared to the parent scores. In both age groups, the 95% limits of agreement are the widest for the Emotions and the Physical Problems scales.

Note: solid blue line = reference line crossing *y*-axis at 0 (no difference); solid red line = mean difference (child/adolescent minus parent score); dashed red line = mean difference +/− 2 standard deviation (95% of responses lie within this range); dashed green line = mean differences +/− 3 standard deviation (*n* = 995 of responses lie within this range).

**Table A3:** Parent–child inter-rater reliability.

QOLIBRI-KID/ADO Scales	Item	Children		Adolescents	
		Item Level Value (95% CI)	Scale Value (95% CI)	Item Level Value (95% CI)	Scale Value (95% CI)
Cognition	Concentration	0.21 (0.094, 0.317) *	0.23 (0.19, 0.277) *	0.44 (0.333, 0.547) *	0.31 (0.268, 0.352) *
	Talking to others	0.19 (0.064, 0.313)		0.27 (0.159, 0.372) *	
	Remembering	0.14 (0.032, 0.247)		0.21 (0.106, 0.323) *	
	Thinking speed	0.16 (0.045, 0.266)		0.19 (0.073, 0.305)	
	Planning	0.11 (0.007, 0.214)		0.13 (0.025, 0.235)	
	Orientation	0.22 (0.079, 0.366)		0.27 (0.12, 0.418) *	
	Decision between two things	0.05 (−0.054, 0.146)		0.13 (0.02, 0.25)	
Self	Appearance	0.13 (0.016, 0.249)	0.14 (0.089, 0.194) *	0.15 (0.033, 0.276)	0.26 (0.202, 0.311) *
	Self-esteem	0.15 (0.036, 0.267)		0.17 (0.048, 0.297)	
	Accomplishment	0.01 (−0.103, 0.128)		0.13 (−0.003, 0.254)	
	Future	0.1 (−0.024, 0.233)		0.27 (0.151, 0.396) *	
	Energy	0.18 (0.072, 0.283)		0.33 (0.208, 0.448) *	
Daily Life and Autonomy	Manage at school	0.26 (0.143, 0.381) *	0.23 (0.183, 0.282) *	0.38 (0.267, 0.503)*	0.32 (0.272, 0.367) *
	Decision making	0.1 (−0.008, 0.201)		0.1 (−0.012, 0.221)	
	Daily independence	0 (−0.134, 0.127)		0.25 (0.067, 0.433)*	
	Getting out and about	0.18 (0.018, 0.34)		0.09 (−0.036, 0.22)	
	Social activities	0.21 (0.087, 0.34) *		0.31 (0.184, 0.436) *	
	Support from others	0.1 (−0.018, 0.225)		0.17 (0.051, 0.287)	
	Ability to move	0.2 (0.059, 0.343)		0.29 (0.147, 0.44) *	
Social Relation-ships	Family relationship	0.17 (0.048, 0.284)	0.2 (0.152, 0.246) *	0.25 (0.139, 0.364) *	0.24 (0.197, 0.287) *
	Relationship with friends	0.24 (0.128, 0.354) *		0.22 (0.094, 0.353)	
	Attitudes from others	0.25 (0.124, 0.369) *		0.19 (0.081, 0.302)	
	Friendships	0.09 (−0.019, 0.199)		0.14 (0.011, 0.278)	
	Open up to others	0.1 (−0.019, 0.228)		0.13 (0.025, 0.23)	
	Demands from others	0.07 (−0.031, 0.165)		0.23 (0.128, 0.338) *	
Emotions	Anger	0.13 (0.019, 0.239)	0.14 (0.087, 0.198) *	0.21 (0.1, 0.324) *	0.14 (0.087, 0.202) *
	Anxiety	0.12 (0.01, 0.223)		0.14 (0.022, 0.248)	
	Sadness	0.13 (0.031, 0.235)		0.03 (−0.077, 0.14)	
	Loneliness	0.11 (−0.012, 0.223)		0.15 (0.027, 0.27)	
Physical Problems	TBI effects	0.22 (0.11, 0.334) *	0.21 (0.166, 0.259) *	0.3 (0.179, 0.425) *	0.27 (0.223, 0.318) *
	Headaches	0.13 (0.013, 0.244)		0.25 (0.132, 0.366) *	
	Pain	0.05 (−0.063, 0.155)		0.1 (−0.007, 0.21)	
	Clumsiness	0.08 (−0.029, 0.183)		0.12 (0.013, 0.231)	
	Seeing/hearing	0.22 (0.086, 0.352)		0.27 (0.158, 0.389) *	
	Other injuries	0.21 (0.09, 0.339) *		0.25 (0.113, 0.39) *	
Total Score			0.09 (0.067, 0.106) *		0.11 (0.095, 0.134) *

Note: Values represent Cohen’s weighed κ statistic; CI = confidence interval, * *p*-value ≤ 0.0001, linear weights were used.

## Appendix C

**Table A4:** Bivariate logistic regression results of child/adolescent characteristics for parent–child-agreement.

Variable *p*-Value ^1^ Unadjusted OR (95% CI)	QOLIBRI-KID/ADO Scales						
	Cognition	Self	Daily Life and Autonomy	Social Relationships	Emotions	Physical Problems	Total Score
Age	**0.001**		**0.004**				
Children vs. Adolescents	0.44 (0.27, 0.72)	1.16 (0.71, 1.90)	0.50 (0.31, 0.80)	0.72 (0.44, 1.18)	1.13 (0.69, 1.84)	0.66 (0.40, 1.09)	0.73 (0.45, 1.19)
Sex							
Female vs. Male	1.12 (0.69, 1.82)	0.94 (0.57, 1.56)	0.78 (0.49, 1.26)	1.14 (0.69, 1.88)	1.57 (0.96, 2.59)	1.33 (0.81, 2.19)	0.82 (0.50, 1.35)
TBI severity							
Moderate or severe vs. Mild	1.27 (0.75, 2.14)	0.84 (0.48, 1.46)	0.90 (0.54, 1.50)	0.64 (0.36, 1.14)	1.20 (0.70, 2.06)	0.69 (0.39, 1.22)	0.69 (0.40, 1.20)
KOSCHI							
3a/b, 4a/b vs. 5a/b	1.01 (0.46, 2.19)	0.89 (0.39, 2.01)	1.04 (0.49, 2.20)	0.64 (0.27, 1.55)	0.73 (0.31, 1.70)	0.90 (0.39, 2.05)	0.51 (0.21, 1.23)
Chronic disease							
Yes vs. No	1.05 (0.60, 1.81)	0.75 (0.42, 1.35)	0.87 (0.51, 1.51)	1.25 (0.71, 2.19)	0.60 (0.33, 1.11)	0.93 (0.52, 1.66)	0.83 (0.47, 1.48)
Pre-TBI mental disorder							
Yes vs. No	0.81 (0.34, 1.92)	0.50 (0.18, 1.38)	0.90 (0.40, 2.07)	1.02 (0.43, 2.44)	0.78 (0.31, 1.92)	1.44 (0.62, 3.34)	0.67 (0.27, 1.66)

Note: Modeling probability: Agreement = Yes. ^1^ Variables in the model: Age, Sex, TBI severity, KOSCHI, Presence of chronic disease, Presence of pre-TBI mental disorder (each variable entered into the model separately). Second category is the reference category. In bold, *p* < 0.05. Only variables fulfilling the entering (0.30) and retaining (0.30) criteria are presented (see Materials and Methods). Final multivariable logistic regression models explaining agreement versus disagreement for each scale and Total. *OR* = odds ratio. *CI* = confidence interval.
